# Medical Treatment and Weight Gain [Response to Letter]

**Published:** 2007-06-15

**Authors:** Astrid Newell, Amy Zlot

**Affiliations:** Oregon Genetics Program, Oregon Department of Human Services, Portland, Ore; Oregon Genetics Program, Oregon Department of Human Services, Portland, Ore

## In Reply:

We appreciate your bringing this important issue to our attention (1). During our review (2), we focused on the role that genetics plays in the development of obesity but did not explore research related to genetics and weight gain associated with medical treatment. A recent article by Muller and Kennedy in the September 2006 issue of *Pharmacogenomics* (3) provides an interesting review of the genetic aspects of weight gain associated with antipsychotic use.

Read the original letter
